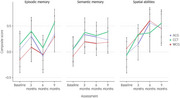# Behavioral and neuronal substrates of serious game‐based computerized cognitive training in cognitive decline related to Alzheimer's Disease

**DOI:** 10.1002/alz70857_102105

**Published:** 2025-12-25

**Authors:** Esther Brill, Alexa Frederike Holfelder, Michael Falkner, Christine R. Krebs, Anna‐Katharine Brem, Stefan Klöppel

**Affiliations:** ^1^ University Hospital of Old Age Psychiatry and Psychotherapy, Bern, Bern, Switzerland; ^2^ ARTORG, University of Bern, Bern, Switzerland; ^3^ King's College London, London, United Kingdom

## Abstract

**Background:**

Subjective cognitive decline (SCD) and Mild Cognitive Impairment (MCI) have predictive value towards Alzheimer's Disease and lend themselves as target for non‐pharmacological preventive interventions such as serious game‐based computerized cognitive training (CCT). However, investigations of CCT show heterogenous results in slowing age‐and disease‐related cognitive decline and adherence to training protocols remain a challenge. The aim of this RCT was to comprehensively evaluate CCTs’ effectiveness, integrating control conditions and neurophysiological as well as blood‐based biomarkers.

**Method:**

In this bi‐centric RCT with parallel groups, 155 participants (mean age 72.2) with cognitive impairment ranging from SCD to MCI were randomized to three arms, comparing CCT to an active control condition (watching documentaries) and a waitlist control condition. Both active arms entailed a three‐month intervention period comprising a total of 60 at‐home sessions (5 sessions per week) and weekly on‐site group meetings. To assess longitudinal effects, the intervention group only completed additional 6 months of at‐home training with monthly on‐site booster sessions. Biological effects were measured by amyloid blood markers and magnetic resonance imaging obtained before and after training.

**Result:**

Significant improvement over time was observed in aggregated domain specific composite scores of episodic and semantic memory and spatial abilities. However, there was no significant interaction between groups and timepoints. Adherence to the training protocol was consistently high across timepoints and groups (4.87 sessions per week). Voxel‐based morphometry revealed no significant changes in grey matter volume following CCT, nor did amyloid levels moderate its effectiveness. Significant cognitive and subjective improvements were observed after long‐term training.

**Conclusion:**

This RCT indicates no specific advantages of a three‐month CCT intervention on cognitive or biological outcomes. However, the consistently high adherence rates observed in this study highlight the importance of designing interventions that align with personal relevance and individual values, particularly in subjects with SCD and MCI who demonstrated high motivation to engage in healthy aging behaviors. Further, positive effects were observed subjectively and after long‐term CCT, warranting the inclusion of CCT in multicomponent interventions.